# Serum zinc concentration in patients with myocardial infarction: a retrospective study

**DOI:** 10.1186/s12872-024-03776-4

**Published:** 2024-02-14

**Authors:** Atsushi Tanita, Shigeto Namiuchi, Kenta Onodera, Shinichiro Sunamura, Tsuyoshi Ogata, Kazuki Noda, Toru Takii

**Affiliations:** Department of Cardiology, Sendai City Medical Center, Sendai Open Hospital, 5-22-1 Tsurugaya, Miyagino-ku, Sendai, 983-0824 Japan

**Keywords:** Zinc, Heart failure, Myocardial infarction, Oxidative stress

## Abstract

**Background:**

Zinc regulates the oxidative stress and inflammatory signaling cascade and affects the development and deterioration of cardiovascular disease. We investigated the prognosis of developing heart failure in patients with myocardial infarction.

**Methods:**

Patients with myocardial infarction (*n* = 243) were divided using the median value of zinc concentration on admission into low (< 66 µg/dL at admission, *n* = 111) and high zinc group (≥ 66 µg/dL at admission, *n* = 132). During follow-up (mean ± SD: 734 ± 597 days; median 691 days), admission due to heart failure was observed in 12 patients: 10 and 2 cases in the low and high zinc groups, respectively.

**Results:**

The risk of admission due to heart failure was significantly higher in the low zinc than in the high zinc group (*P* = 0.0043). Relative to the high zinc group, the hazard ratio for admission due to heart failure was 15.7 (95% confidence interval 1.11–221, *P* = 0.042) via adjusted Cox proportional hazards analysis. Even after propensity score matching, the risk of admission due to heart failure was significantly higher in the low zinc than in the high zinc group (*P* = 0.048).

**Conclusion:**

Low serum zinc concentration may be a risk factor for admission due to heart failure after myocardial infarction.

## Background

Zinc is an essential trace element and a component of several metalloenzymes with redox capacity; therefore, it has a critical role in maintaining human health, especially regarding anti-oxidative stress and anti-inflammation. Zinc deficiency is thought to affect vascular and cardiac cellular dysfunction [1]. Although the standard value of serum zinc concentration is 80–130 µg/dL, a decrease in serum zinc levels has been observed in patients with myocardial infarction (MI) [2] and has been reported earlier to be associated with the development of fatal arrhythmia and poor prognosis after MI [3]. Moreover, it has been indicated that zinc supplementation reduced ventricular arrhythmia [4] and attenuated left ventricular remodeling [5] in experimental MI models in rats. However, there is a report that zinc concentration was not associated with mortality in patients with acute MI [6]. Whether serum zinc concentrations affect the prognosis of patients after MI is still controversial. Heart failure (HF) development is one factor that strongly determines the prognosis in patients with MI. We investigated the effects of zinc concentration on the prognosis of patients with MI, particularly from the perspective of developing HF.

## Methods

### Study population

In this retrospective study, we investigated zinc concentrations in 250 patients with acute MI, according to the universal definition, from January 2016 to December 2021. The cases with in-hospital death (7 patients) were excluded, and the remaining 243 patients were divided into two groups using the median value of zinc concentration on admission to compare the risk of re-admission due to HF in the two groups.

### Protocol

We analyzed patient characteristics such as age, sex, body mass index (BMI), coronary risk factors, patient status on admission, and medication after hospitalization. Diagnoses of hypertension, diabetes mellitus, and dyslipidemia were obtained from the patients’ medical records or histories from previous medical therapy.

Serum zinc concentration was measured on admission. Blood samples for measuring serum levels of zinc were collected in 10-mL vacutainers and centrifuged at 1700 *g* for 5 min. The zinc concentration was measured by a colorimetric assay using 2- (5-Bromo-2-pyridylazo) -5- [N-n-propyl-N- (3-sulfopropyl) amino] phenol (5-Br-PAPS). The analysis was performed on a Canon TBA-nx360 (Canon Medical Systems Corporation, Tochigi, Japan).

The outcome of this study was hospital admission due to HF. Medical charts were retrospectively reviewed to obtain data on in-hospital and post-discharge outcomes, which had been assessed based on the patient’s history of regular visits to the hospital after discharge, contact with the patient’s family, and contact with family doctors.

This study conformed to the principles outlined in the Declaration of Helsinki. In addition, this study was approved by the Sendai City Medical Center’s ethical committee (number 2022-0061) and all the patients provided written informed consent.

### Statistical analysis

Continuous data are presented as mean ± standard deviation (or as median and interquartile range) and were compared using the Student’s *t*-test. Categorical data are presented as percentages and were compared using the Chi-square test. We constructed the following three Cox proportional hazards regression models: an unadjusted model, an age- and sex-adjusted model, and a fully adjusted model. In the fully adjusted model, we included the zinc level and 15 variables that were considered influencing factors for admission due to HF [age, sex, BMI (kg/m^2^), walking independently, previous MI, previous admission due to HF, hypertension, hemoglobin (g/dL), creatinine (mg/dL), C-reactive protein (mg/dL), Killip class ≥ 2 on admission, left ventricular ejection fraction (LVEF, %), peak creatine kinase (U/L), and prescription at discharge (angiotensin converting enzyme inhibitor [ACEI] or angiotensin II receptor blocker [ARB] and β-blocker). Furthermore, using propensity scores for zinc levels, which were estimated for each participant using the 15 baseline covariates above, 62 pairs of participants with low and high zinc groups were matched, and Kaplan–Meier estimates were compared using log-rank tests to evaluate the associations between the zinc level and the risk of admission due to HF before and after propensity score matching. Values of *P* < 0.05 were considered statistically significant. We used JMP software (JMP version 14.2.0, SAS Institute, Cary, North Carolina, USA) for statistical analysis. Data were at least 91% complete for all variables examined. The most common missing covariate was HbA1c (*n* = 21, 8.6% missing) and total cholesterol (*n* = 15, 6.2% missing).

## Results

### Patients’ characteristics and laboratory findings

Two hundred and forty-three patients were investigated retrospectively. The mean patient age was 67 ± 15 years, and 74% of the patients were male. Serum zinc concentration on admission was 67 ± 14 µg/dL, and the median value was 66 µg/dL. The patients were divided into two groups: the low zinc group (< 66 µg/dL at admission, *n* = 111) and the high zinc group (≥ 66 µg/dL at admission, *n* = 132). Table 1 compares the baseline characteristics and laboratory findings at admission between patients in the low and high zinc groups. The patients in the low zinc group were older and had lower hemoglobin and higher C-reactive protein levels than those in the high zinc group. There were no significant differences in the history of MI, admission due to HF, and stroke. Table 2 presents the patient status upon admission and in-hospital management. Patients in the low zinc group had higher Killip class and significantly lower LVEF than those in the high zinc group. Both groups had similar proportions of patients with emergent coronary angiography and primary percutaneous coronary intervention (PCI). There was no significant difference in peak creatine kinase levels. In medication upon discharge, the proportion of patients prescribed ACEI/ARB was lower, and that of patients prescribed β-blocker was higher in the low zinc group than the high zinc group.

**Table 1:** Patient characteristics and laboratory findings

	Overall cohort (*n* = 243)	Low zinc group (*n* = 111)	High zinc group (*n* = 132)	*P*
Age (years)	67 ± 15	70 ± 16	65 ± 14	0.013
Male sex	179 (74%)	78 (70%)	101 (77%)	0.27
Body mass index (kg/m^2^)	24.2 ± 3.9	23.8 ± 3.8	24.6 ± 4.1	0.12
Walking independently	220/242 (91%)	93/110 (85%)	127/132 (96%)	0.0017
Dyslipidemia	138/243 (57%)	56/111 (50%)	82/132 (62%)	0.067
Diabetes mellitus	80/243 (33%)	40/111 (36%)	40/132 (30%)	0.34
Hypertension	147/243 (60%)	74/111 (67%)	73/132 (55%)	0.071
Previous MI	17/243 (7%)	10/111 (9%)	7/132 (5%)	0.26
Previous HF admission	1/243 (0.4%)	0/111 (0%)	1/132 (1%)	0.36
Previous stroke	23/243 (9%)	13/111 (12%)	10/132 (8%)	0.27
Chronic hemodialysis	1/243 (0.4%)	1/111 (0.4%)	0/132 (0%)	0.27
Chronic AF	17/243 (7%)	10/111 (9%)	7/132 (5%)	0.26
Zinc (µg/dL)	67 ± 14	55 ± 8	77 ± 10	< 0.0001
Hemoglobin (g/dL)	14.1 ± 2.0	13.4 ± 2.2	14.6 ± 1.7	< 0.0001
Creatinine (mg/dL)	0.91 ± 0.42	0.97 ± 0.57	0.88 ± 0.23	0.089
Creatine kinase (U/L)	142 [80, 350]	182 [95, 490]	126 [75, 290]	0.12
C-reactive protein (mg/dL)	0.2 [0.1, 0.5]	0.2 [0.1, 1.7]	0.1 [0.1, 0.3]	< 0.0001
BS (mg/dL)	172 ± 74	178 ± 82	166 ± 67	0.19
HbA1c (%)	6.4 ± 1.4	6.5 ± 1.6	6.4 ± 1.2	0.53
Total cholesterol (mg/dL)	204 ± 54	201 ± 62	207 ± 45	0.36
LDL-cholesterol (mg/dL)	126 ± 42	122 ± 45	129 ± 40	0.26
HDL-cholesterol (mg/dL)	46 ± 11	46 ± 11	47 ± 12	0.26
Triglyceride (mg/dL)	125 [79, 183]	104 [70, 171]	132 [96, 203]	0.93

MI, myocardial infarction; HF, heart failure; AF, atrial fibrillation; BS, blood sugar; HbA1c, hemoglobin A1c; LDL, low density lipoprotein; HDL, high density lipoprotein

**Table 2:** Patient status upon admission and in-hospital management

	Overall cohort (*n* = 243)	Low zinc group (*n* = 111)	High zinc group (*n* = 132)	*P*
Time from the onset to hospital (hours)	2.3 [1.1, 6.2]	2.9 [1.3, 6.5]	2.0 [1.0, 6.1]	0.46
STEMI	162/243 (59%)	74/111 (64%)	9/7132 (69%)	0.41
Killip class (I/II/III/IV)	199/18/11/15	85/12/8/6	114/6/3/9	0.062
Killip class ≥ 2	44/243 (18%)	26/111 (23%)	18/132 (14%)	0.048
LVEF (%)	56 ± 10	54 ± 10	57 ± 10	0.014
Emergent CAG	231/243 (95%)	104/111 (94%)	127/132 (96%)	0.37
Primary PCI	211/243 (87%)	96/111 (86%)	115/132 (87%)	0.88
IABP	17/243 (7%)	10/111 (9%)	7/132 (5%)	0.26
PCPS	1/243 (0.4%)	1/111 (1%)	0/132 (0%)	0.27
Peak CK (U/L)	1246 [437, 2992]	1198 [389, 2914]	1504 [496, 3095]	0.84
Medication				
ACEI/ARB	203/243 (84%)	87/111 (78%)	116/132 (88%)	0.047
β-blocker	175/243 (72%)	88/111 (79%)	87/132 (66%)	0.021
MRA	12/243 (5%)	4/111 (4%)	8/132 (6%)	0.38
Statin	213/243 (88%)	95/111 (86%)	118/132 (89%)	0.37

STEMI, ST elevated myocardial infarction; LVEF, left ventricular ejection fraction; CAG, coronary angiography; PCI, percutaneous coronary intervention; IABP, intra-aortic balloon pumping; PCPS, percutaneous cardiopulmonary support; CK, creatine kinase; ACEI, angiotensin converting enzyme inhibitor; ARB, angiotensin receptor blocker; MRA, mineralocorticoid receptor antagonist

### Clinical outcomes

**Fig. 1:**
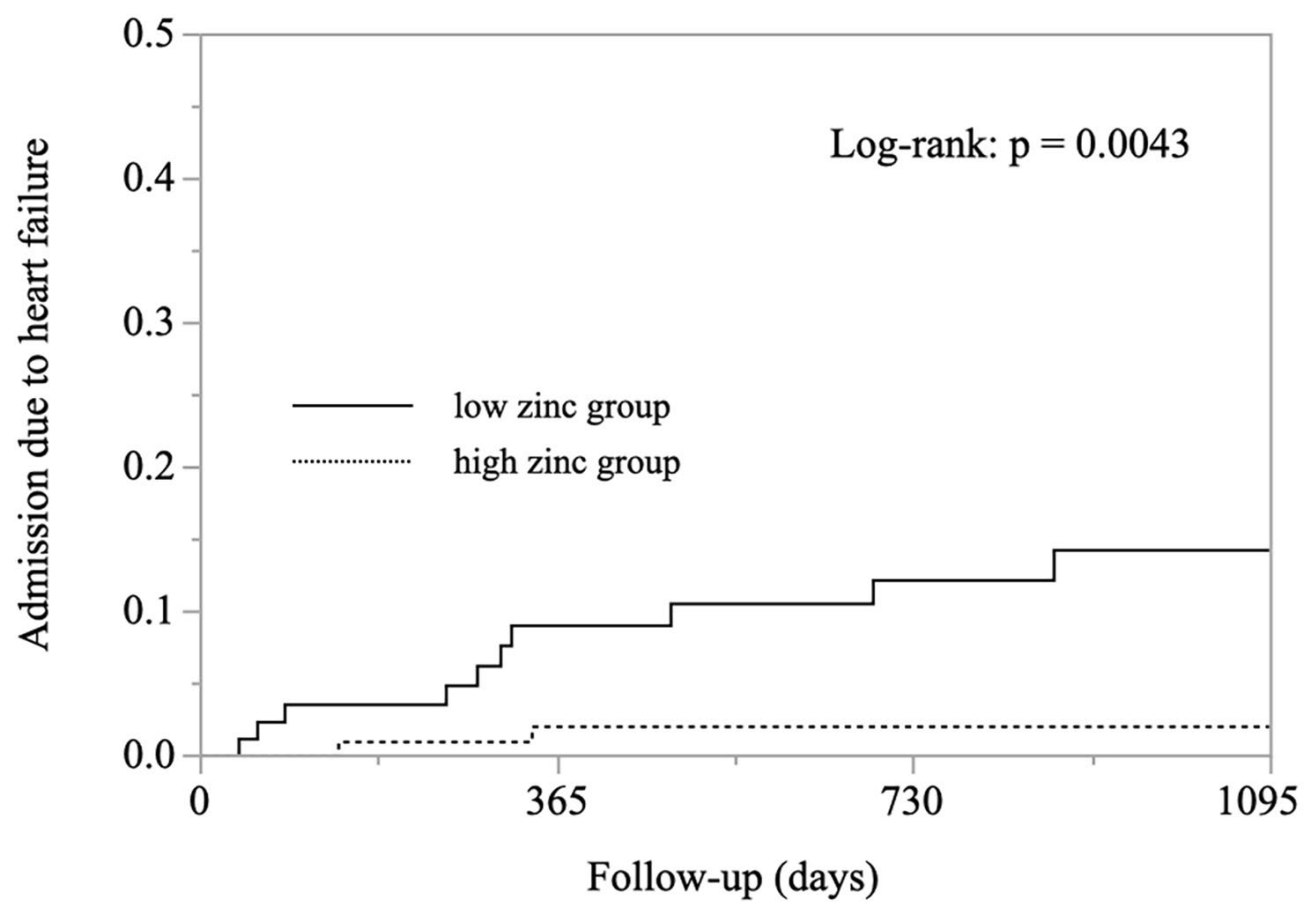
Kaplan–Meier curves for admission due to heart failure in patients in the low zinc (solid line) and high zinc (dotted line) groups

During the follow-up periods (mean ± SD: 734 ± 597 days; median 691 days), admission due to HF was observed in 12 patients: 10 cases in the low zinc group and 2 cases in the high zinc group. Kaplan–Meier analysis revealed that the risk of admission due to HF was significantly higher in the low zinc group than in the high zinc group (*P* = 0.0043) (Fig. 1). Table 3 presents the results of Cox proportional hazards models for admission due to HF. In the unadjusted model, the hazard ratio for admission due to HF was 6.75 (95% confidence interval [CI] 1.48–30.8) in the low zinc group, compared to that in the high zinc group. The analogous hazard ratio was 4.76 (95% CI 1.04–21.8) in the age and sex-adjusted model in the low zinc group.

**Table 3:** Cox proportional-hazard models for HF admission in the zinc groups

	Hazard ratio	95% CI	*P*
Unadjusted			
High zinc group	1.0 (reference)		
Low zinc group	6.75	1.48–30.8	0.014
Age and sex-adjusted			
High zinc group	1.0 (reference)		
Low zinc group	4.76	1.04–21.8	0.044
Fully adjusted			
High zinc group	1.0 (reference)		
Low zinc group	15.7	1.11–221	0.042

HF, heart failure; CI, confidence interval

**Fig. 2:**
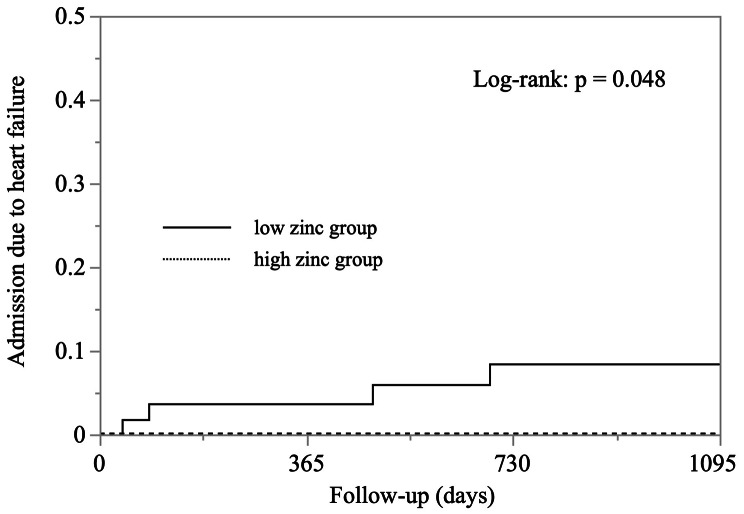
Kaplan–Meier curves after propensity score matching for admission due to heart failure in patients in low zinc (solid line) and high zinc (dotted line) groups

Moreover, in the fully adjusted model, the risk of admission due to HF was significantly greater in patients in the low zinc group than in the high zinc group (hazard ratio 15.7, 95% CI 1.11–221, *P* = 0.042). LVEF and previous HF history were also significant independent variables for admission due to HF. After propensity score matching using 15 variables, Kaplan–Meier analysis revealed that the risk of admission due to HF was significantly higher in the low zinc group than in the high zinc group (*P* = 0.048) (Fig. 2).

## Discussion

Zinc is an essential nutrient for human health and has anti-oxidative stress and anti-inflammatory functions. Low zinc levels are frequently found in patients with cardiovascular disease. HF is a progressive systemic illness accompanying oxidative stress in multiple tissues and pro-inflammatory phenotypes, and zinc deficiency may adversely affect the development and progression of HF. Low zinc levels may also lead to the progression of the atherosclerotic process due to a reduction in antioxidant and anti-inflammatory capacity caused by low serum zinc levels [1]. A meta-analysis showed that the serum zinc levels in HF patients were significantly lower than those in control subjects [7], and a recent report has demonstrated that HF patients with low zinc levels have a higher mortality rate [8]. Low zinc levels have also been reported in patients with MI [2]. Many factors contribute to zinc deficiency, including reduced dietary intake, reduced absorption, increased uptake in stressed tissues, and increased excretion [9].

In the present study, there were many patients with poor general condition in the low zinc group. The patients in the low zinc group were older and had lower hemoglobin levels. These differences were quite obvious, suggesting that poor general health condition may have a role in patient prognosis. However, the significant effect of zinc levels on the risk of HF was confirmed even in analyses adjusted these variables, suggesting that zinc level was associated with the risk of readmission due to HF, independent of age and hemoglobin concentration. In addition, the low zinc group had a more elevated Killip class and reduced LVEF than the high zinc group, suggesting the presence of HF status in certain patients. Low zinc levels in those patients may have been due to inadequate intake and/or malabsorption of nutrients from intestinal edema associated with congestion. Zinc levels are influenced by many factors including diet, digestion, absorption, inflammation, and immunity. It is not possible to exclude the role of these factors and consider the effects of zinc levels in isolation. Serum zinc concentration may be considered as one of the factors that indicate patients’ general health condition. Furthermore, inflammatory cytokines, such as interleukin-1, monocyte chemoattractant protein-1, and tumor necrosis factor-𝛼 are known to be elevated in the acute phase of myocardial infarction [10, 11]. The hypercatabolic state caused by increased cytokines may also influence the increased demand for zinc. The above-mentioned indicators of inflammation, which are considered to be more accurate, were not measured in this study. However, C-reactive protein levels were significantly higher in the low zinc group compared to in the high zinc group. These findings on general indicators of inflammation suggest that inflammatory state may be involved in the poor prognosis of patients in the low zinc group.

It is not yet clear how low zinc levels are associated with the development of HF. One possibility is that many patients with a low serum zinc concentration are in poor general condition, indirectly increasing the incidence of HF. In addition, it is necessary to consider the possibility that low zinc levels cannot adequately suppress oxidative stress and inflammatory responses, leading to the development of HF. In terms of HF after MI, an excessive inflammatory response could promote infarct expansion and exacerbate left ventricular (LV) remodeling after MI affecting the infarct healing process [12]. Inhibition of the exaggerated inflammatory response may be beneficial to prevent LV remodeling [13]. A previous study reported that the antioxidant effect of zinc comprises its role as an endogenous inhibitor for the entry of prooxidant Ca^2+^ and its regulation of antioxidant defense genes activated metal-responsive transcription factor. The use of zinc as an antioxidant may protect vulnerable cardiomyocytes under assault from neurohormonal activation and spare the myocardium from adverse structural remodeling [14].

A recent study of patients with MI undergoing primary PCI reported no significant difference in mortality between low and high zinc groups [6]. However, previous studies have suggested that low zinc levels adversely influence the prognosis in patients with MI [3] or HF [8]; this result suggests that the effects of zinc concentration on mortality after MI may be masked by the larger effect of primary PCI. Primary PCI is one of the factors that strongly influences the prognosis of patients with MI, including the development of HF. Several studies in Japan reported the incidence of HF within 2 years after MI to be approximately 3–4% [15–17], while in the present study it was 6.5%. These incidences are relatively low compared with those reported in the Western countries [18, 19], probably due to the high rate of reperfusion therapy. The presence or absence of primary PCI may also be related to the influence of zinc concentration on the prognosis after MI. A recent study on patients with MI undergoing primary PCI reported no significant difference in mortality between low and high zinc group [6]. However, a previous study from the pre-PCI era suggested that low zinc levels adversely influence the prognosis in patients with MI [3]; this result suggests that the effects of primary PCI on the mortality after MI are greater than those of zinc concentration, which may be masked. Our study included approximately 10% of cases in which primary PCI was not performed and the outcome was admission due to HF. These differences may have led to prognostic differences between the low and high zinc groups. Our research revealed that the risk of admission due to HF was significantly higher in patients with low zinc levels, and the result was the same even after adjusting for differences in patient backgrounds using Cox proportional hazard analysis and propensity score matching.

Although there is no consensus on whether zinc supplementation can prevent the incidence of HF and improve the prognosis of patients, there are several reports that zinc supplementation was beneficial in experimental MI models in rats [4, 5, 20]. In humans, there is a recent report of a case with new-onset HF in which remarkable improvement was observed after zinc administration [21]. Moreover, it has been reported that zinc infusion improved cardiac function in patients with intestinal malabsorption and zinc-deficient cardiomyopathy [22]. However, further studies are needed to determine whether zinc supplementation benefits all HF cases.

This study has a few limitations. First, this study was retrospective, observational, and conducted at a single center. Further, the results were obtained from a relatively small number of patients. The most important limitation is that the results were obtained from a small number of HF events, and the actual number of patients with HF after MI was 12 cases across approximately two years. Moreover, zinc levels were measured at only one point, and changes in zinc levels over time were not followed. Changes in therapy after discharge may have affected the zinc levels and the development of HF. Additional studies with larger sample sizes are needed to validate the conclusions of this study and determine whether anti-oxidative stress and anti-inflammatory intervention through zinc supplementation could improve the prognosis of patients after MI.

## Conclusions

Low serum zinc concentration may be a risk factor for admission due to HF after MI.

## Data Availability

The data presented in this study are available on reasonable request from the corresponding author.

## Abbreviations

MI: Myocardial infarction
HF: Heart failure
BMI: Body mass index
LVEF: Left ventricular ejection fraction
PCI: Percutaneous coronary intervention
ACEI: Angiotensin converting enzyme inhibitor
ARB: Angiotensin II receptor blocker
CI: Confidence interval
